# Biomarker role in assessing imaging needs for mild cranial trauma (BRAIN-CT): study protocol for a single-center, randomized controlled trial

**DOI:** 10.3389/fneur.2025.1692163

**Published:** 2026-01-09

**Authors:** Ali Tfaily, Ariana Chacon, Tianwen Ma, Jonathan Ratcliff, Randi Smith, Hassan Saad, Andrew Reisner, David Gimbel, Kevin Wang, Firas Kobeissy, Jonathan A. Grossberg, Ali M. Alawieh

**Affiliations:** 1 Department of Neurosurgery, Emory University School of Medicine, Atlanta, GA, United States; 2 Department of Biostatistics and Bioinformatics, Emory University Rollins School of Public Health, Atlanta, GA, United States; 3 Department of Emergency Medicine, Emory University School of Medicine, Atlanta, GA, United States; 4 Department of Surgery, Emory University School of Medicine, Atlanta, GA, United States; 5 Center for Neurotrauma, Multiomics and Biomarkers (CNMB), Morehouse School of Medicine, Atlanta, GA, United States

**Keywords:** mild traumatic brain injury (mTBI), TBI biomarkers, GFAP, UCH-L1, clinical trial

## Abstract

**Introduction:**

Mild traumatic brain injury (mTBI) accounts for a significant proportion of emergency department (ED) visits, but current diagnostic protocols often lead to overuse of computed tomography (CT) imaging, despite low diagnostic yield. The BRAIN-CT trial evaluates the impact of rapid access to TBI biomarkers on decision-making for cranial imaging in patients with mTBI.

**Methods and analysis:**

This randomized controlled trial will enroll 350 adult patients aged 18–85 years presenting with suspected mild head injury (Glasgow Coma Scale 13–15) within 24 h of trauma. Participants will be randomized into two arms: (1) a biomarker-published group where ED providers receive real-time results of the i-STAT® TBI Cartridge test (detecting GFAP and UCH-L1), and (2) a biomarker-blinded group where results are withheld. The primary outcome is the proportion of patients undergoing CT imaging. Secondary outcomes include hospital length of stay, cost, neurological outcomes, and biomarker correlation with imaging findings. Analysis will involve chi-squared testing, logistic regression, and predictive modeling.

**Clinical trial registration:**

clinicaltrials.gov, identifier: NCT06932588.

## Introduction

Mild traumatic brain injury (mTBI) is the most prevalent form of head trauma presenting to emergency departments (EDs) globally, accounting for over 90% of the more than 5 million cases reported annually in the United States (1). About 82% of patients with mTBI undergo computed tomography (CT) imaging in the ED to exclude intracranial hemorrhage or other serious brain injuries, particularly in patients presenting with risk factors (1–3). However, this approach has led to the routine overuse of CT scans, with up to 90% of scans returning negative results and fewer than 1% of cases necessitating neurosurgical intervention (4–7). This overutilization contributes to increased healthcare costs, prolonged ED stays, and unnecessary radiation exposure (4–8).

The substantial economic burden associated with the acute evaluation of mTBI highlights the need for more efficient diagnostic pathways. In recent years, blood-based biomarkers have emerged as promising tools to improve triage decisions in patients with suspected mTBI. Several blood-based biomarkers have been studied in the context of mTBI including glial fibrillary acidic protein (GFAP), ubiquitin carboxy-terminal hydrolase L1 (UCH-L1), S100 calcium binding protein-B (S100B) among others (9–13). However, GFAP and UCH-L1 have both showed the highest sensitivity in predicting intracranial abnormalities on CT of the head in prospective observational cohorts (9, 12, 14). GFAP, an intermediate filament protein expressed by astrocytes, rises in the bloodstream over several hours following astroglial injury (15), whereas UCH-L1, a neuronal enzyme involved in protein turnover, increases more rapidly after neuronal damage (10). These complementary temporal profiles underpin the rationale for their combined use, which has demonstrated high sensitivity and negative predictive value (NPV) for detecting intracranial lesions in mTBI patients, with strong potential for ruling out significant intracranial pathology (9, 16). Table 1 summarizes the major clinical studies involving the utility of GFAP and UCH-L1 in mTBI.

**Table 1:** Clinical studies involving the utility of GFAP and UCH-L1 in mTBI.

Study	Sample size	Population	Age	Sample type	BM	Thresholds	Sens/Spec/NPV
Bazarian et al., 2018 (12)	1,920	Suspected mTBI (GCS 14–15)	≥18 years	Serum	GFAP, UCH-L1	GFAP: 22 pg./mL; UCH-L1: 327 pg./mL	Sens: 97.3%; Spec: 36.7%; NPV: 99.5%
Legramante et al., 2024 (13)	130	Suspected mTBI (GCS 13–15)	≥18 years	Serum	GFAP, UCH-L1	GFAP: 35 pg./mL; UCH-L1: 400 pg./mL	Sens: 100%; Spec: 27.6%; NPV: 100%
Papa et al., 2016 (14)	584	Trauma patients with and without mTBI	≥18 years	Serum	GFAP, UCH-L1	AUC for GFAP and UCH-L1	Diagnostic ranges of AUC were 0.80 to 0.97 for GFAP and 0.31 to 0.77 for UCH-L1 over 7 days.
Karamian et al., 2025 (15)	5,921	Suspected mTBI (GCS 13–15)	≥18 years	Serum/Plasma	GFAP, UCH-L1	GFAP: 4 pg./mL; UCH-L1: 64 pg./mL	GFAP: Sens: 98%, Spec: 15%, NPV: 97%; UCH-L1: Sens: 99%, Spec: 18%, NPV: 99%
Papa et al., 2012 (16)	96	Mild and moderate TBI	≥18 years	Serum	UCH-L1	UCH-L1: 0.09 ng/mL	Sens: 100%; Spec: 21%; NPV: 100%
Abbott i-STAT Whole Blood Test (17)	~250 (validation study)	Suspected mTBI (GCS 13–15)	≥18 years	Whole Blood	GFAP, UCH-L1	GFAP: 65 pg./mL; UCH-L1: 360 pg./mL	Sens: 96.5%; NPV: 96.5%

BM, biomarkers; mTBI, mild traumatic brain injury; GCS, Glasgow Coma Scale; Sens, sensitivity; Spec, specificity; NPV, negative predictive value.

The growing body of clinical evidence informed the development of the i-STAT TBI Plasma test (Abbott, Inc.), a point-of-care diagnostic tool that simultaneously measures GFAP and UCH-L1 concentrations. This test received FDA clearance (17) based on results from the ALERT-TBI study, which enrolled nearly 2,000 patients and demonstrated a sensitivity of 97.6% and an NPV of 99.6% for ruling out traumatic intracranial injury (9). Using the approved thresholds for both biomarkers, and despite high sensitivity for detecting intracranial injury, the specificity remains low (<40%) indicating that these tests are to be used to rule out intracranial pathology in mTBI rather than to rule in Table 1. Secondary analyses from ALERT-TBI further suggested that incorporating the i-STAT TBI test into ED workflows could reduce CT scan usage by approximately 40% (18). Both tested biomarkers where noted to be persistently elevated at least for 24 h after injury (19). Despite these promising findings, adoption of TBI biomarker testing in routine ED and trauma care remains limited, and integration into clinical algorithms is still evolving. Traditionally, TBI guidelines have routinely involved recommendations for CT head imaging for patients presenting with mTBI; however, emerging guidelines from Europe and recent recommendations from the NIH-NINDS TBI classification and nomenclature initiative have started to incorporate biochemical biomarkers in management of mild TBI (20–22).

The Biomarker Role in Assessing Imaging Needs for Mild Cranial Trauma (BRAIN-CT) trial is a prospective, randomized, double-blind clinical trial designed to evaluate the impact of real-time biomarker testing, combined with provider education, on CT utilization in patients with suspected mTBI. The study will employ the i-STAT Alinity instrument and i-STAT TBI Test (Abbott, Inc.), which provides quantitative measurement of the GFAP and UCH-L1 biomarkers in the whole blood samples at the bedside within 15 min, facilitating timely decision-making without impeding ED throughput. In addition to the primary outcome of CT scan reduction, the trial will assess secondary outcomes including the predictive value of biomarkers for intracranial pathology, length of ED stays, radiation exposure, hospital costs, and short-term functional outcomes.

This paper presents the rationale, design, and methodology of the BRAIN-CT trial and outlines the anticipated clinical implications of integrating blood-based biomarker testing into acute mTBI management.

## Methods and analysis

### Study objective

The BRAIN-CT trial investigates whether providing emergency-department clinicians with rapid GFAP and UCH-L1 results, via the point-of-care i-STAT TBI cartridge, changes imaging behavior in adults with mild traumatic brain injury. The primary objective (Table 2) is to compare the proportion of patients who receive a head CT scan between a biomarker-published arm and a standard-care (biomarker-blinded) arm; we hypothesize that real-time access to GFAP and UCH-L1 levels will lower the CT scan rate compared with standard management. To address this objective, we have designed a prospective, randomized, open-label, blinded-endpoint (PROBE) adaptive trial that will enroll 350 participants, randomized 1:1 to biomarker-published versus biomarker-blinded arms using permuted blocks stratified by age group, biological sex, race, and baseline Canadian CT Head Rule risk category (Supplementary Table 1). Frequent interim analyses with prespecified efficacy and futility boundaries will permit early stopping if criteria are met.

**Table 2:** Primary and secondary objectives of the BRAIN-CT trial.

Objective	Category	Objective description	Outcome measured
Primary	–	Evaluate whether real-time access to point-of-care TBI biomarker results (GFAP, UCH-L1) reduces the use of cranial CT imaging.	Proportion of patients undergoing head CT.
Secondary (clinical impact) two-arm comparisons	Clinical decision-making	Assess the impact of biomarker result access on physician decision-making for CT use.	CT imaging timing and provider rationale.
	Efficiency and cost	Determine whether biomarker use reduces ED/hospital length of stay and healthcare costs.	LOS, resource use, hospital charges. Perform a formal ICEA of biomarker testing versus standard care.
	Patient outcomes	Compare short-term outcomes between arms (neurological deterioration, readmissions, GOS, GCS).	GOS-E/GCS at 6 months and 1-year follow-up, neurological symptoms.
	Radiation exposure	Quantify radiation dose reduction per patient between arms.	Directly measure a key safety benefit.
	Disparity analysis	Identify patient subgroups (e.g., by age, injury mechanism, comorbidity) where biomarkers influence decision-making.	Stratified CT usage, subgroup analysis of impact.
	Safety monitoring	Monitor for adverse outcomes from missed injuries in biomarker-guided arm.	Adverse events, unexpected deterioration, delayed diagnoses.
	Time-to-imaging decision	Compare time from triage to CT decision between arms.	Evaluate whether biomarker access accelerates or delays decision-making.
	Disposition flow	Evaluate differences in ED-to-disposition pathways with vs. without biomarker data.	ED throughput times, floor/ICU admissions.
Secondary (validation) full cohort as single arm	Diagnostic accuracy	Determine sensitivity, specificity, NPV, and PPV of biomarkers vs. CT findings for intracranial lesions.	Biomarker levels vs. CT results.
	Kinetic profiling	Evaluate the kinetics and temporal profile of GFAP/UCH-L1 across 0, 8, 16, and 24 h.	Serial biomarker levels, time-to-peak and decay curves.
	Predictive modeling	Build multivariable models using biomarkers and clinical data to predict imaging-positive patients.	ROC curves, AUC, logistic regression outputs.
	Triage integration	Assess biomarker utility in supplementing current BIG criteria for TBI triage.	Sensitivity/specificity when combined with BIG criteria.
	Outcome prediction	Evaluate whether biomarker levels predict functional outcomes or neurologic decline.	Correlation with GOSE score, readmission, or symptom progression.
Secondary (implementation) only published cohort	Implementation feasibility	Assess provider adherence to biomarker interpretation guidelines in the biomarker-published arm.	Understand clinical adoption barriers and fidelity to protocol including provider and patient variables
	Patient perception	Evaluate patient understanding and satisfaction with biomarker-driven decision-making.	Gain insights into trust and acceptance of non-imaging diagnostics.

TBI, traumatic brain injury; CT, computed tomography; ED, emergency department; LOS, length of stay; ICEA, incremental cost-effectiveness analysis; GCS, Glasgow Coma Scale; GOS, Glasgow Outcome Scale; GOSE, Glasgow Outcome Scale-Extended; ICU, intensive care unit; NPV, Negative predictive value; PPV, Positive predictive value; ROC, Receiver Operating Characteristic; AUC, Area under the curve; BIG, Brain Injury Guidelines.

Secondary objectives are divided into three categories as summarized in Table 2; clinical impact, validation studies, and implementation studies. Clinical impact aims will explore whether access to biomarkers impacts the use of hospital resources, shortens length of stay, reduces radiation exposure, and predicts clinical outcomes. Validation objectives aim to replicate published data on the prognostic potential of the biomarkers studied on CT imaging and to study the timeline of biomarker changes in the blood after injury. Finally, implementation objectives study the predictors of incorporating guidelines into the decision making by providers.

### Study design and methods

**Figure 1:**
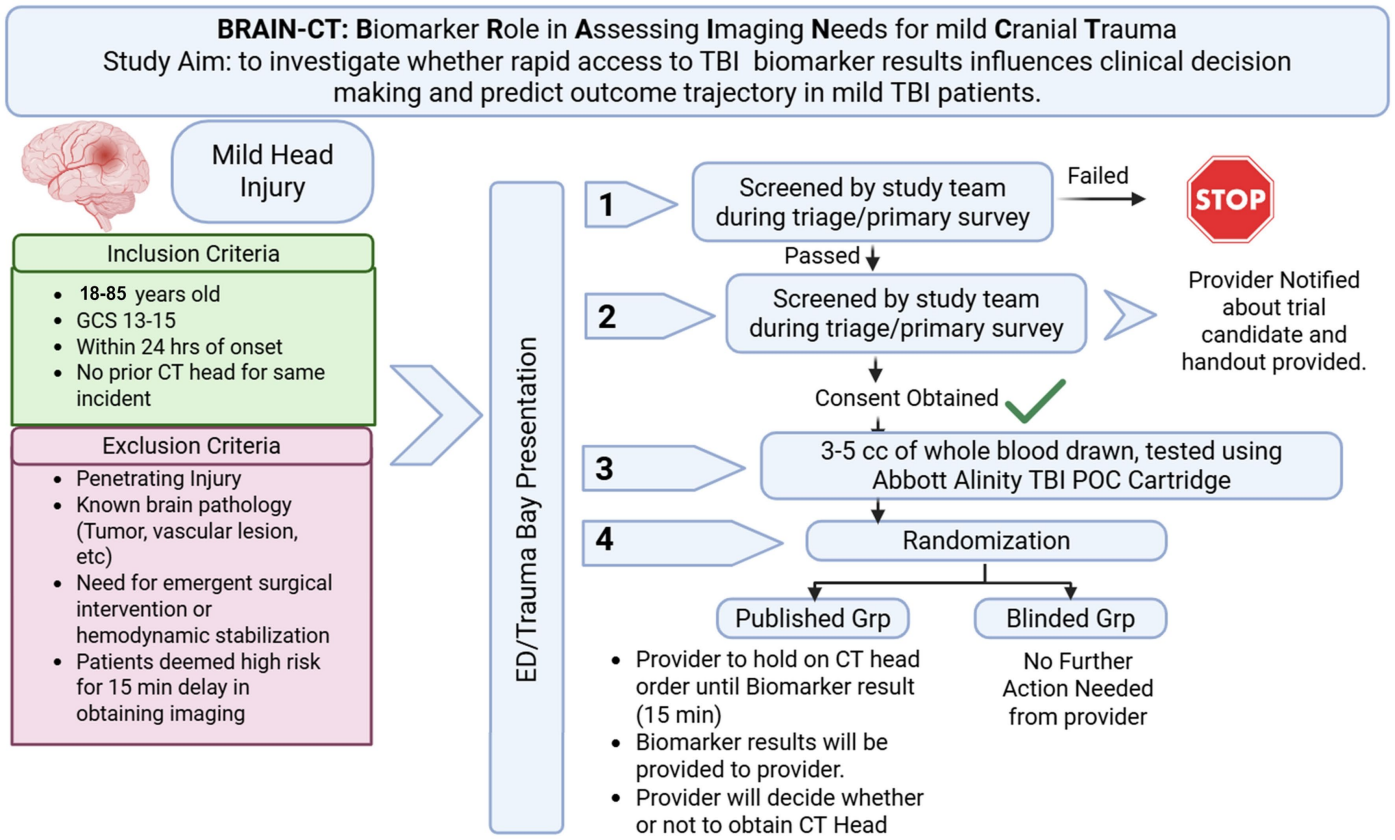
Study algorithm for enrollment, testing and reporting.

The BRAIN-CT trial is a single-center prospective, randomized, open-label, blinded endpoint study conducted in the ED of Grady Memorial Hospital, a level I trauma center. After completion of initial screening, 350 adults aged 18–85 years will be randomized 1:1 to either a biomarker-published arm (real-time GFAP and UCH-L1 values released to clinicians coupled to education on biomarker data) or a biomarker-blinded arm (standard care without biomarker disclosure). The study flow chart is provided in Figure 1. Allocation uses computer-generated permuted blocks stratified by age (3 Strata: 18–30, 31–50, 51–85 years), biological sex, race (2 Strata: Black, Not Black), Glascow Coma Scale (GCS) (2 Strata: 15, 13–14) and baseline Canadian CT Head Rule risk category (3 Strata: High Risk, Medium Risk, None) to balance key factors in routine decision making. The maximum sample size remains 350 participants for the initially planned cohort. Outcome adjudicators are masked to treatment assignments. Given the pre-existing FDA-approval of the use of the TBI biomarker testing in mild-TBI, the study is deemed no more than minimal risk to participants and a data safety and monitoring board was not required. This study is registered with clinicaltrials.gov (Identifier: NCT06932588), and the study was approved by the Institutional Review Board at Emory University and the Research Oversight Committee at Grady Memorial Hospital.

### Inclusion and exclusion criteria

The study inclusion criteria include adults aged 18–85 years old presenting with mild TBI, defined as Glasgow Coma Scale (GCS) score 13–15, within 24 h of injury and without prior head imaging for the same incident.

For definition of timing of injury, this data will be obtained from emergency medical personnel reports, witness reports, reported history from family members or companions, and information from outside facility records if the patient was transferred. If no specific time of injury was determined, the last known normal time will be used. If the last known normal time was within 24 h of presentation, the subject will be included. If the last known normal time is over 24 h, and the exact time of incidence is not available, the patient will not be included.

Key exclusion criteria include:

- Subjects with penetrating head injury.
- History of known brain abnormality including tumor, cerebrovascular malformation, recent brain surgery (within 6 months), prior head injury (within 6 months).
- Need for emergent surgical intervention for brain or alternate body organ injury.
- Need for emergent bedside procedures for hemodynamic or orthopedic stabilization.
- Patients deemed high risk for decline by the provider prohibiting the 15 min delay in obtaining imaging needed for biomarker testing.
- Minors, pregnant women and prisoners.

### Pre-randomization procedures

Screening of eligible patients will be performed by study personnel via real-time tracking of ED dashboard for new admissions or transfers. Triage notes and evaluations will be reviewed for primary eligibility based on age, type of injury, reported GCS, timing of injury and whether existing head imaging is present. If subjects pass screening, they will be approached for review of detailed inclusion and exclusion criteria and for consent process. Written informed consent (from the patient or legally authorized representative) is obtained before any study-specific activities. Providers are notified about patient enrollment and requested to hold off on obtaining CT head imaging until randomization and biomarker data is available. Randomization variables will then be obtained, and subjects will be randomized using an automated algorithm implemented in RedCap into published or blinded biomarker groups.

### Procedures for blinded biomarker arm

For the blinded arm, no further changes are requested from the provider, and a venous blood sample is then collected for point-of-care GFAP and UCH-L1 measurement using the i-STAT TBI cartridge; results are withheld from clinicians. The patient will then be followed up during the hospitalization for reviewing provider decisions, imaging data, clinical outcomes, and disposition as listed in the outcome section.

### Procedures for the published biomarker arm

For the published arm, providers are requested to pause imaging orders until biomarker data is available which requires around 15 min delay. When biomarker data is available, these are printed and provided timely to the provider along with the cut-off for an elevated test. The cut-off used are 65 pg./mL for GFAP and 360 pg./mL for UCH-L1. The provider will also be handed a refresher education on the published data on the negative predictive value (NPV) and positive predictive value (PPV) of the test. The decision whether to pursue a CT of the head will be left to the provider and patient discussion considering the biomarker data. A test is considered non-elevated only if both biomarkers were below the pre-defined cut-off.

### Biomarker testing

Venous blood will be collected by licensed phlebotomists and will be immediately loaded on the iSTAT cartridges as per manufacturer’s instructions. Analysis will be performed as provided by the manufacturer and by dedicated technicians who have been trained and approved by the manufacturer to perform the test. A training log is maintained with the study document for all training sessions and refreshers provided. For every batch/lot of cartridges, independent control testing using manufacturer’s provided controls is performed for quality control.

### Post-randomization in-hospital assessment

After allocation, participants are followed throughout their ED stay and – if admitted– for the duration of hospitalization. Study coordinators prospectively capture whether a head-CT is performed, its timing and radiology results; emergency-department and inpatient length of stay; any neurological deterioration or other adverse events; and final disposition or admission status.

### Post-discharge follow-up

Study staff will conduct structured telephone interviews at 6 months and 12 months after the ED visit to ascertain persistent or late-presenting neurologic symptoms, unplanned healthcare utilization (ED visits, hospital readmissions, outpatient imaging), and any delayed diagnoses of intracranial injury. Future contact is approved by subjects at the time of consent. When participants cannot be reached after three call attempts, electronic health-record review and contact of designated surrogates are attempted to complete the data set. Designated surrogates are individuals determined by the subject to be their emergency contact for the study for future contact and are named at the time of informed consent.

### Primary efficacy analysis

The primary endpoint is the proportion of patients undergoing a head CT scan which will be reported as percentage of total patients. The key hypothesis:

- H_0_ (null): There is no difference in CT head scan rates between the published biomarker and blinded biomarker arms.
- H_1_ (alternative): The published biomarker arm has a significantly lower CT scan rate compared to the blinded biomarker arm.

To evaluate the hypothesis, we will perform univariate analysis using a Pearson’s chi-square test followed by a pre-specified logistic regression model that adjusts for stratification/randomization variables (e.g., site, age, GCS score category), providing an adjusted odds ratio (aOR) and 95% confidence interval (CI). A statistically significant result will be defined with a two-tailed *p*-value of less than 0.05, or adjusted odds ratios for predicting CT head acquisition with a 95% confidence interval below 1.0. In the event the *p*-value approaches the significance threshold (e.g., p between 0.05 and 0.10), results will be interpreted with caution, and effect size estimates will be emphasized to assess the practical impact of the observed differences.

For subgroup analysis across randomization strata, we will use the same testing as primary hypothesis. Then, we will formally test for heterogeneity of treatment effect using interaction terms in the logistic regression models. A *p* < 0.05 for the interaction will be considered evidence of differential efficacy across subgroups (given the exploratory nature of interaction testing).

### Safety analysis

Safety analysis will be evaluated by assessing the rate of delayed diagnosis of significant intracranial findings that require intervention and will be compared between the two groups using a Pearson’s Chi-squared test. The time to CT head imaging will also be compared between two groups using a two-sample *t*-test or Wilcoxon rank sum test depending on the normality assumption.

### Secondary analyses

Relevant secondary analyses reported in Table 2 will be studies as described below:

- Assess the impact of biomarker result access on physician decision-making for CT use (Time to obtaining CT head, *t*-test).
- Hospital Length of stay in published versus blinded group (*t*-test).
- Hospital costs for published versus blinded group (Incremental cost effectiveness analysis).
- GCS and GOS-E comparison in published versus blinded groups at 6 and 12 months (Ordinal logistic regressions).
- Compare rates of discharge, floor admission, and ICU admission between the published and blinded groups (logistic regression for each outcome, taking admission GCS and age as covariates).
- Within all patients, define the sensitivity and specificity for predicting GOS-E scores at 6 and 12 months for biomarker test as well as when combined with clinical variables and BIG criteria (ROC analysis).
- Within the published group, study predictors of adherence to biomarker-based guidelines among providers (logistic regression).

### Sample size justification

Based on preliminary data from published studies, we defined a clinically significant impact to be attained at 15% reduction in the rate of CT head imaging in the published group, assuming 70% baseline CT utilization (23). Adjusting for an alpha of 5%, a total of 300 evaluable patients will be required (150/arm) to achieve at least 80% power to reject the null hypothesis. To account for patient withdrawal (change in exam before blood draw, leaving against medical advice, etc.), we will plan to target a maximum of 350 subjects enrolled.

### Safety monitoring

All adverse events from consent through hospital discharge–neurological deterioration, unplanned neurosurgical intervention, escalation of care, or death–are recorded prospectively; serious AEs (death, life-threatening event, or event resulting in prolonged hospitalization) are reported to the IRB and sponsor within 24 h. A blinded investigator adjudicates event relatedness to study procedures. An independent Medical Monitor reviews cumulative safety data quarterly and at each interim analysis, with authority to recommend protocol modifications or early termination if predefined neurologic severe adverse events thresholds are exceeded (Table 3).

**Table 3:** SPIRIT guidelines.

Timepoint	Study period				
	Enrolment	Allocation	Post-allocation	Follow up	Close-out
	t1	t2	t3	t4	t5
Enrolment					
Eligibility screen	X				
Informed consent	X				
Baseline assessment (GCS, labs)	X				
Randomization		X			
Interventions					
Biomarker testing		X	X		
Biomarker result communicated		X (if applicable)	X		
Standard care			X		
Assessments					
Head CT (if performed)			X		
Length of stay			X		
Adverse events monitoring			X		
Outcome adjudication (blinded)			X		
6 months follow-up (phone)				X	
12 months follow-up (phone)					X

GCS, Glasgow Coma Scale; Labs, Laboratories; CT, computed tomography.

### Rationale for early biomarker use

GFAP and UCH-L1 enter the bloodstream minutes to hours after mild traumatic brain injury and, when measured on the handheld i-STAT TBI cartridge, yield quantitative results in ≈ 15 min. Large prospective cohorts and FDA-clearance data show that the combined assay has ≥96% sensitivity and >99% NPV for predicting CT-positive intracranial injury within 24 h of trauma, permitting safe imaging deferral in biomarker non-elevated cases (12, 19, 24). Because standard decision rules (e.g., the Canadian CT Head Rule, institutional common practices) still lead to CT scanning in up to 70% of emergency-department patients, embedding real-time GFAP and UCH-L1 testing into the initial evaluation offers an evidence-based, objective tool to reduce unnecessary radiation, crowding, and cost while maintaining diagnostic safety (25).

### Rationale for randomization

Randomization ensures that known and unknown confounders–such as injury mechanism, clinician decision-making patterns, and patient comorbidities–are evenly distributed between the biomarker-published and biomarker-blinded arms, thereby allowing unbiased estimation of the causal effect of real-time biomarker disclosure on head-CT utilization. Stratification by age, biological sex, race, GCS score and Canadian CT Head Rule risk category further improves balance across prognostic variables. Randomization is essential in this trial because CT decisions are inherently subjective, and without allocation concealment, selection bias or preferential imaging decisions could distort observed differences. The 1:1 parallel-group design also facilitates straightforward intention-to-treat analysis and preserves statistical validity in interim monitoring.

## Discussion

### Generalizability

The BRAIN-CT clinical trial has been designed to improve diagnostic decision-making for patients with mild traumatic brain injury (mTBI) and leverage the advantages of new biomarker technology in the acute setting. EDs face a critical challenge: balancing the need to detect clinically significant intracranial injuries with the imperative to reduce unnecessary imaging, radiation exposure, and healthcare costs. The broad inclusion criteria were selected to target a population of mTBI patients who are most frequently subjected to head CT scanning despite low rates of positive findings. This population represents a substantial portion of ED visits and an area in which diagnostic innovation is urgently needed. Exclusion criteria were guided by three principles: (1) In whom might biomarker-guided imaging deferral be safe and effective (2) In whom might this approach introduce risk or diagnostic uncertainty and (3) The ethical obligation to generate rigorous data that can elevate the standard of care in mTBI.

### Expected impact of the proposed trial

We are currently unaware of Level I, evidence-based protocols that use blood biomarkers to guide cranial imaging after mTBI. The BRAIN-CT trial is designed to test whether a point-of-care GFAP and UCH-L1 assay, performed within 24 h of injury, can safely reduce CT utilization while maintaining clinical safety and lowering healthcare costs. Previous efforts to limit unnecessary imaging–such as the Canadian CT Head Rule–have demonstrated high sensitivity for detecting clinically significant injuries (2, 6), yet their clinical adoption has been inconsistent. Factors contributing to this include workflow variability, clinician preference, and perceived medico-legal risk (7). The BRAIN-CT trial addresses these limitations by offering an objective, rapid biochemical assessment that is integrated into routine ED workflows. Coupled with rigorous trial methodology, including randomization, blinded outcome adjudication, and cost-effectiveness analysis, BRAIN-CT is positioned to provide critical randomized data on the feasibility, safety, and clinical impact of biomarker-guided imaging decisions, helping to inform future multicenter studies and guideline development.

### Dissemination

The trial results will be disseminated through publications in peer-reviewed journals, presentations at scientific conferences, and media channels. The datasets generated during the study will be available upon reasonable request.

### Limitations

This study has limitations that should be considered when interpreting its findings. First, the BRAIN-CT trial is conducted at a single, high-volume, urban Level I trauma center, which may limit generalizability to other healthcare environments. Patterns of emergency-department workflow, clinician comfort with mTBI management, and baseline CT utilization vary widely across institutions. As such, reductions in imaging achieved in this setting may not fully translate to community hospitals, rural trauma centers, or systems with differences in patient demographics, resource availability, or medico-legal practices. Future multicenter studies will be necessary to determine whether biomarker-guided imaging algorithms perform consistently across diverse clinical settings. Second, the study relies on a single biomarker platform—the i-STAT TBI cartridge—with manufacturer-defined thresholds for GFAP and UCH-L1. Although these biomarkers have strong sensitivity and negative predictive value for intracranial injury, the low specificity of these biomarkers may still result in some unnecessary imaging, and biomarker performance may differ in populations with comorbidities (e.g., polytrauma, chronic neurological disease, intoxication) or in cases involving repeated injuries.
