# Supplementary material for: Biomarker role in assessing imaging needs for mild cranial trauma (BRAIN-CT): study protocol for a single-center, randomized controlled trial
Source: Front Neurol. 2026 Jan 9;16:1692163. doi: 10.3389/fneur.2025.1692163 (PMC12827124; doi:10.3389/fneur.2025.1692163)
Supplement: Supplementary file 1 [file Table_1.DOCX]

**Supplementary Material**

**Supplementary Table 1. Description of the Canadian CT head Rule.**

| **Supplementary Table 1. Canadian CT Head Rule (CCHR)** | | |
| --- | --- | --- |
| **Category** | **Criteria** | **Clinical Significance** |
| **Intended Population** | Adults (≥16 years) with **minor head injury** defined as: • Blunt trauma to the head AND • Witnessed loss of consciousness, amnesia, or disorientation AND • Initial GCS 13–15 | Target group in whom the CCHR decision tool applies |
| **Risk Groups** | | |
| **High-Risk Group** (for **neurological intervention**) | • GCS < 15 at 2 hours after injury • Suspected open or depressed skull fracture • Any sign of basal skull fracture: Battle’s sign, raccoon eyes, CSF otorrhea/rhinorrhea, hemotympanum • ≥2 episodes of vomiting • Age ≥ 65 years | **Mandates CT** due to high risk of clinically important brain injury requiring neurosurgical intervention |
| **Medium-Risk Group** (for **clinically important brain injury on CT**) | • Amnesia before impact ≥ 30 minutes • Dangerous mechanism of injury (pedestrian struck; occupant ejected; fall from ≥3 ft or ≥5 stairs) | **CT recommended** due to increased risk of intracranial injury |
| **Low-Risk Group** | • Absence of factors above | **CT optional** |
